# Not All Orbitopathy Is Graves’: Discussion of Cases and Review of Literature

**DOI:** 10.3389/fendo.2017.00184

**Published:** 2017-07-31

**Authors:** Neeraja Boddu, Maliha Jumani, Vibhor Wadhwa, Gitanjali Bajaj, Fred Faas

**Affiliations:** ^1^Endocrinology, University of Arkansas for Medical Sciences, Little Rock, AR, United States; ^2^Radiology, University of Arkansas for Medical Sciences, Little Rock, AR, United States

**Keywords:** differential of thyroid-associated orbitopathy, Graves’ orbitopathy, orbital lymphoma, sarcoid orbitopathy, orbitopathy

## Abstract

**Introduction:**

Graves’ orbitopathy is the extra thyroidal manifestation of Graves’ disease and the most common cause of exophthalmos. It is also known as thyroid-associated orbitopathy (TAO) as it occasionally occurs in euthyroid or hypothyroid patients with chronic thyroiditis. 5% of patients with Graves’ orbitopathy can be euthyroid or hypothyroid as they have low titers of anti-thyrotropin-receptor antibodies, which are difficult to detect in some assays. Orbitopathy has also been seen in a small percentage of patients with Hashimotos thyroiditis. The eye involvement in Graves’ is frequently bilateral and symmetric. These patients pose few diagnostic difficulties when the ocular findings occur concomitantly with the thyroid disease. However, when unilateral and asymmetric ocular findings occur with normal or mildly abnormal thyroid function tests, alternate etiologies should also be pursued. We aim to discuss some conditions like sarcoidosis, lymphoma, orbital pseudotumor, and orbital malignancy that mimic TAO.

**Cases:**

Three patients were referred to us with concern for Graves’ orbitopathy. After further work-up, we diagnosed the first patient with specific orbital myositis from sarcoidosis. Our second patient had CD10-positive B-cell lymphoma. Our third patient had orbitopathy likely secondary to Hashimotos or orbital pseudotumor.

**Conclusion:**

Our cases and discussion describe some other conditions that clinically mimic TAO and the importance of pursuing further work-up for accurate diagnosis when presentation of orbitopathy is atypical.

Graves’ orbitopathy also referred to as thyroid-associated orbitopathy (TAO) is the extra thyroidal manifestation of Graves’ disease and the most common cause of exophthalmos. It is an immune disorder causing inflammation and expansion of orbital fat and muscle. It is seen in 25–50% of patients with Graves’ disease and hyperthyroidism. Occasionally, it occurs in those with Graves’ disease with no evident clinical symptoms or biochemical thyroid abnormality (1). Few cases have also been reported to occur in patients with Hashimoto’s thyroiditis (2). Clinical features of TAO include exophthalmos, lid retraction, periorbital swelling, ophthalmoplegia, and chemosis (3). Diagnosis is based on clinical features and may be supported by abnormal thyroid function tests and positive thyroid antibodies (4). Mostly, eye signs are bilateral but unilateral changes may occur in about 15% of patients and sometimes precede the onset of Graves’ (5). Although, this disorder has strong correlation with TSH receptor antibodies, some cases have been reported with antibody negativity (6). When endocrinologists receive referrals for euthyroid patients with negative thyroid antibodies with orbitopathy suspicious for Graves’, they need to be aware of other conditions that may mimic Graves’ orbitopathy. Some of these disorders are sarcoidosis, lymphoma, orbital pseudotumor, and orbital malignancy. We present three cases of orbitopathy referred to our Endocrine clinic for suspected TAO and a review of literature on the differential diagnosis of the same.

## Case 1

**Figure 1:**
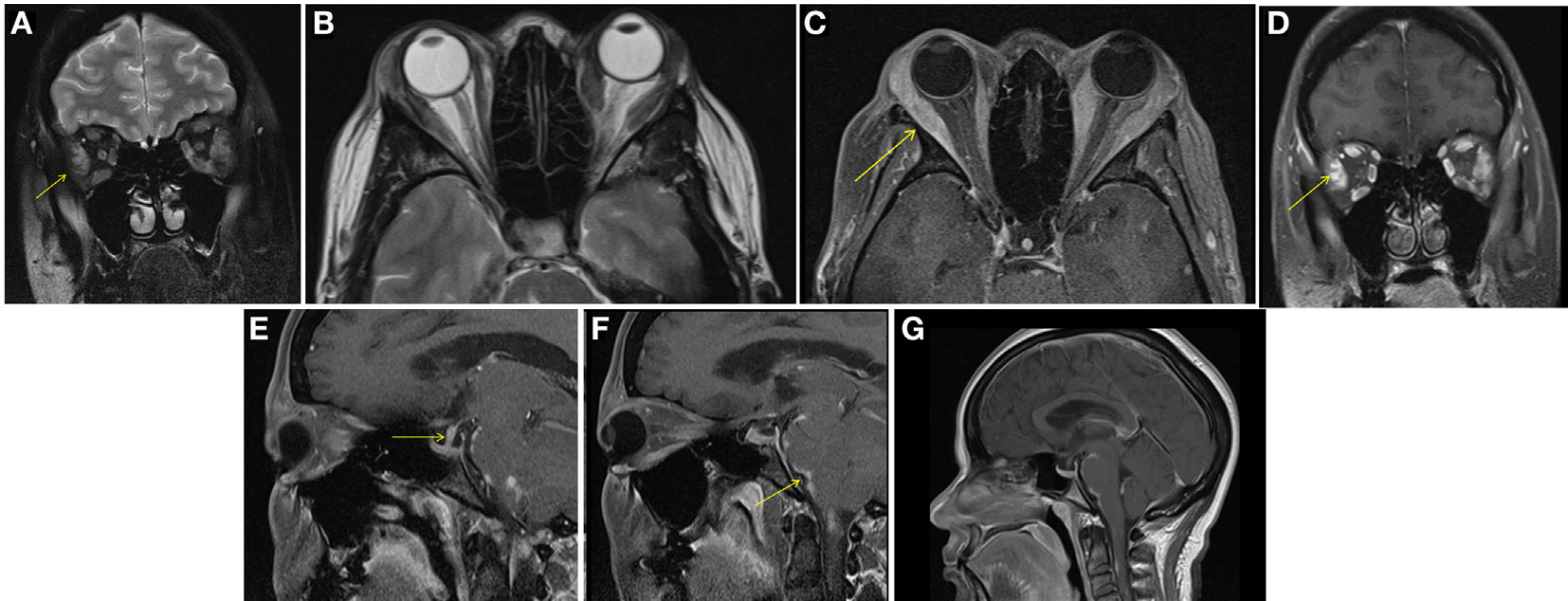
Case 1. Diffuse enlargement and enhancement of muscle bellies and anterior tendinous insertions of bilateral extraocular muscle on coronal and axial T2 **(A,B)** and fat suppressed post contrast T1 **(C,D)** orbital images. Involvement of the tendons and lateral rectus muscle makes Graves’ orbitopathy less likely. Additional findings, which favored the diagnosis of sarcoidosis included nodular leptomeningeal enhancement **(F,G)** with thickening of the pituitary stalk **(E)**.

A female aged 40–50 years presented with a 2-month history of intermittent headaches, heat intolerance, fatigue, right facial numbness, double vision, and periorbital swelling. On exam, she had mild thyromegaly with a nodular texture and no palpable cervical adenopathy. Eye exam showed proptosis, periorbital edema, and diplopia. TSH was 5.11 μIU/ml (0.450–4.500 μIU/ml) with a normal free T4 0.88 ng/dl (0.82–1.77 ng/dl). Thyroid peroxidase (TPO) antibodies were elevated at 356 IU/ml (0–34 IU/ml). TSH receptor antibodies (TRAb) were 0.51 IU/L (0.00—1.75 IU/L) and Thyroid stimulating immunoglobulin (TSI) 51% (0–139%). Thyroid ultrasound showed a multinodular goiter with right thyroid lobe enlargement. CT scan of the orbits showed bilateral asymmetric enlargement of extraocular muscles (EOMs) with suspicion for Graves’ orbitopathy. MRI of the orbits confirmed EOM enlargement; largest in bilateral lateral rectus, left medial rectus, and left inferior rectus. Closer review of MRI images also showed lacrimal gland enlargement, leptomeningeal enhancement of the surface of the brain and spinal canal, and thickened infundibulum raising concern for sarcoidosis (Figure 1). Subsequently, a 1 cm right thyroid nodule was biopsied showing granulomatous changes consistent with sarcoidosis. During the course of her followup, she also developed complete heart block and required pacemaker placement. Patient was started on prednisone 20 mg daily with marked improvement in her visual symptoms. Repeat MRI of the brain after steroids showed marked improvement in size of EOMs, lacrimal glands, and leptomeningeal disease. Repeat thyroid ultrasound showed decrease in the size of the goiter with no discrete nodularity, microcalcifications, or other concerning features. This patient had orbital, neurologic, and cardiac involvement with sarcoidosis. She had no pulmonary nodules or mediastinal findings on further imaging.

## Case 2

**Figure 2:**
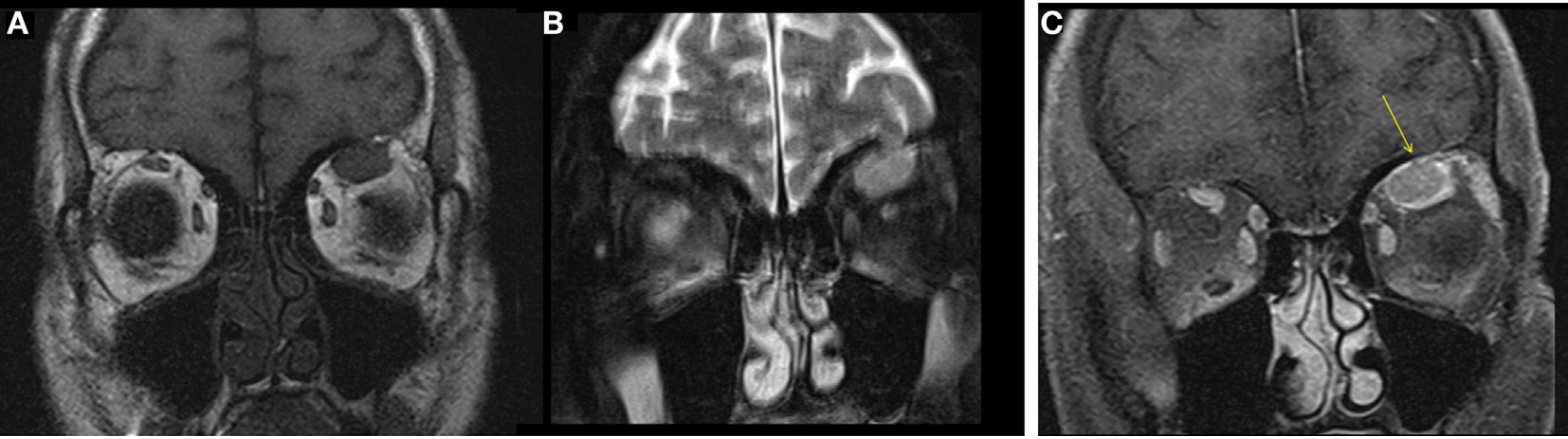
Case 2. T1 hypointense and T2 hyperintense mildly enhancing mass in the left superior rectus muscle seen on coronal T1 **(A)**, fat saturated T2 **(B)**, and post contrast fat saturated T1 **(C)** images of the orbits. Solitary muscle involvement especially of the superior rectus makes thyroid orbitopathy an unlikely etiology.

A male aged 50–60 years was referred with left eye proptosis progressively getting worse for 2 months. He denied visual changes, headache, eye pain, fatigue, diaphoresis, palpitations, tremor, or weight changes. Visual field testing was normal. Exophthalmos was noted in the left eye with forward displacement of 21 mm compared to 17 mm on the right. MRI of the brain and orbits showed an enhancing circumscribed mass within the superior aspect of the left orbit measuring 4.2 cm in greatest dimension with thickening of the anterior tendinous portion of superior rectus. The globe was displaced inferiorly and laterally. The lacrimal glands showed no enlargement (Figure 2). Also noted incidentally was a right sided pituitary macroadenoma measuring 17 mm × 21 mm and extending to the right cavernous sinus with left shift of the pituitary infundibulum but no significant compression of optic chiasm. Differentials for the extraocular muscle involvement were felt to be orbital pseudotumor, unusual asymmetric enlarged muscle in Graves’ disease or an infiltrative tumor involving the muscle. Biochemical evaluation for thyroid and pituitary was within normal range. Subsequently, the patient underwent transsphenoidal resection of non-secretory pituitary macroadenoma. He continued to have left eye proptosis and was given empiric steroid taper with temporary resolution of proptosis and decrease in muscle swelling on MRI. However, symptoms recurred when steroids were tapered. Fine needle aspiration (FNA) of left levator palpebrae muscle was performed twice and was negative for malignancy. Due to continued symptoms, patient underwent left lateral orbitotomy with bone removal and biopsy of the lesion. Biopsy showed CD10-positive B-cell lymphoma. This was an indolent follicular/marginal zone lymphoma involving the superior rectus muscle. Patient received radiation therapy with improvement in symptoms.

## Case 3

**Figure 3:**
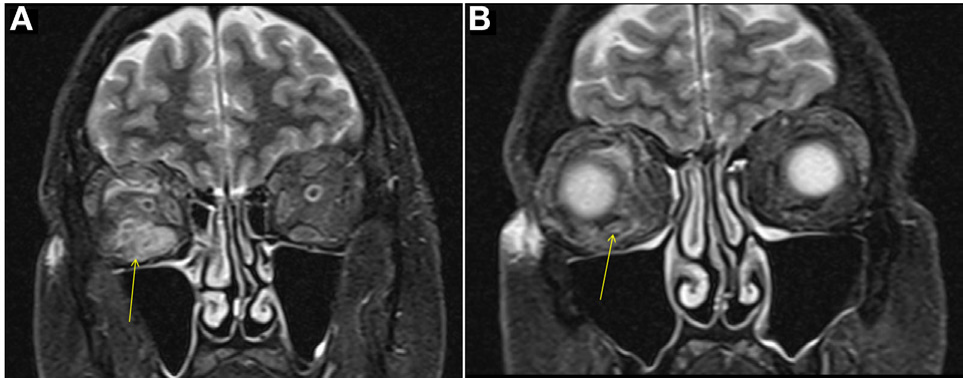
Case 3. Coronal T2W MRI images showing enlargement of the right inferior rectus muscle [arrow in **(A)**] with surrounding fat stranding, features similar to thyroid-associated orbitopathy (TAO). Focal sparing of the anterior tendinous insertion [arrow in **(B)**] is another common finding of TAO.

A male aged 50–60 years was referred with right eye proptosis and double vision of 1-year duration. His only symptom was fatigue. No thyromegaly was noted. He had diplopia on both vertical and horizontal gaze. Exophthalmos was noted, 25 mm on right and 20 mm on left. Biochemical work-up showed a TSH of 149 μIU/ml. TSI and TSH receptor antibodies were negative, but TPO antibodies were positive. Given biochemical confirmation of hypothyroidism, patient was started on levothyroxine. An MRI was obtained for evaluation of orbital disease and it showed mild diffuse mid-belly enlargement and T2 hyperintensity of EOMs bilaterally. There was moderate enlargement of the right inferior rectus muscle (Figure 3). His imaging findings were concerning for TAO because of involvement of the right inferior rectus muscle and diffusely hazy orbital fat. Focal sparing of the tendon of the inferior rectus further supported this. He was started on steroid eye drops followed by oral steroids. Orbital swelling improves while on steroids but recurs when the dose is tapered. FNA of inferior rectus was performed while patient was on steroids. The specimen was small and inconclusive and showed mild inflammation only.

## Discussion

Graves’ orbitopathy has an incidence rate of 16 women and 3 men per 100,000 (7). It occasionally occurs in euthyroid or hypothyroid patients with chronic thyroiditis. The 5% of patients with Graves’ orbitopathy who are euthyroid or hypothyroid generally have low titers of TRAbs, which are difficult to detect in some assays (3, 7). It may occur in a small percentage of patients with Hashimoto’s thyroiditis as well (6, 8). Most speculated mechanism causing Graves’ orbitopathy is that autoantibodies are formed against thyrotropin receptors that are found in both the thyroid gland and orbit. This causes activation of T lymphocytes with subsequent release of inflammatory mediators (9). The muscles are infiltrated with inflammatory cells and hyaluron causing enlargement of the EOMs and an increase in the orbital fat volume (10). Ultimately, collagen deposition leads to fibrosis. The involvement is frequently bilateral and symmetric (11). Patients with Graves’ Orbitopathy pose few diagnostic difficulties when ocular findings occur concomitantly with the thyroid disease. But unilateral or inconclusive ocular features in the absence of objective evidence of thyroid dysfunction need pursuance of alternate etiologies (12). We aim to discuss some conditions that mimic TAO and our cases provide examples of the potential pitfalls in delineating TAO from these other conditions.

## Diagnostic Difficulties

Clinically eyelid retraction, proptosis, chemosis, restrictive ophthalmopathy with diplopia, and in worst cases, compressive optic neuropathy are seen with TAO (1). Radiographically extraocular muscle enlargement sparing the tendons is seen (4). Extraocular muscle involvement is frequently bilateral with more than one muscle group involved and with regular borders. However, none of these features are specific. Other etiologies should be considered when the presentation is atypical. These other conditions could be inflammatory, neoplastic, infectious, vascular, or neuromuscular (Table 1). Often, autoimmune thyroiditis may also occur in conjunction with other systemic autoimmune conditions like sarcoidosis, Sjogren’s disease, systemic sclerosis, systemic lupus erythematosus (SLE), and rheumatoid arthritis, which may further complicate the diagnosis (13). Clinically, time of onset of symptoms and disease progression might provide some clue to alternate diagnosis. Orbital cellulitis is acute and rapidly progressive. Orbital myositis from systemic causes may be subacute in onset over days or weeks and may be associated with pain. Neoplastic involvement may be painless and restrictive and occur more gradually over weeks or months. It also may present as mass effect. Very often orbital biopsy has to be offered to confirm a diagnosis and properly tailor the treatment plan in these cases (12).

**Table 1:** Differential diagnosis of Graves’ orbitopathy.

Differential diagnosis of Graves’ orbitopathy
Specific orbital inflammation ~5% A Sarcoidosis B Systemic lupus erythematosus C Crohn’s disease D Scleroderma Orbital pseudotumor/non-specific orbital inflammation ~40% Neoplasms ~20–40% A Lymphomas B Benign tumors—hamartomas, primary granulosa cell tumors, rhabdomyomas, liposarcomas C Metastases—melanoma, breast, gastrointestinal, and lung carcinomas Others ~10–15% A Vascular malformations B Infections C Neuromuscular dysfunction

### Specific Orbital Inflammation and Myositis

This is a less common cause of orbitopathy accounting for ~5% cases compared to non-specific inflammation, which accounts for about 40% cases in a reported study (12). It occurs from systemic inflammatory causes like sarcoidosis, SLE, Crohn’s disease, or scleroderma. Our first patient presented with this etiology.

Sarcoidosis is a multisystem granulomatous disease characterized histologically by non-caseating epithelioid-cell granulomas in affected tissue. It presents in the third or fourth decades and more commonly affects blacks. 30–35% of all sarcoidosis patients may have ophthalmic involvement; 7–19% of these patients have systemic disease along with ocular symptoms but others could have isolated orbital disease with lacrimal gland involvement and very rarely extraocular muscle involvement (14, 15). In these cases, incorrect diagnosis of Graves’ orbitopathy may be made especially if concomitant thyroid function test abnormality is present. The unilateral or bilateral proptosis in sarcoidosis results from retro-orbital infiltration by sarcoid tissue. The imaging findings are non-specific and include enhancement of EOMs such as in Graves’ disease, infiltrative mass identical to orbital pseudotumor or lymphoma and rarely thickening, nodularity of sclera (16, 17). Involvement of other intracranial structures and presence of parenchymal, leptomeningeal, dural, or skull findings may be clue to diagnosis. Sarcoid granuloma in the thyroid is extremely rare, with a prevalence of 4% in autopsied patients with systemic sarcoidosis. Hypothyroidism may occur through extensive infiltration by epithelioid granulomas, which was the case in our patient (18). Also a significantly higher prevalence of clinical hypothyroidism and Graves’ disease has been observed in female patients with sarcoidosis than in control subjects. It is important to test thyroid function and thyroid antibodies in these patients (19).

Systemic lupus erythematosus, which is a collagen vascular disease resulting in multisystem involvement and vasculitis may also cause Bilateral orbital involvement and specific myositis in few case reports (20). Other symptoms like rash, photosensitivity, and arthritis may give a clue to diagnosis. This condition also usually responds to high-dose steroids and disease modifying antirheumatic drugs like hydroxychloroquine. Crohn’s disease is another granulomatous inflammatory condition of the bowels causing orbital disease in about 13%. It may present as iritis, scleritis, optic neuritis, and retinal involvement. Bilateral involvement of orbital muscles in these patients has been reported. Again excellent response to steroids is seen. The pathogenesis is speculated to be immune complexes, which have affinity for EOMs (21). Scleroderma is another chronic autoimmune condition causing thickening and fibrosis of skin that may also cause inflammation, fibrosis, and extraocular muscle restriction like Graves’ (12).

### Primary or Secondary Neoplasms

This is a reported cause in about 20–40% of cases with non-thyroid orbitopathy (12). Our second patient had presented with progressively worsening unilateral proptosis. He did not have symptoms of hyperthyroidism on presentation and no prior history of the same. Thyroid antibodies were negative and he had radiologically confirmed unilateral disease. This along with the fact that he only had transient improvement in symptoms with steroids prompted us to look for alternative diagnoses. Indeed, a biopsy revealed lymphoma.

Orbital lymphoma can mimic TAO as it may originate from or infiltrate the EOMs (22). Conjunctival disease is frequent and can be an isolated finding in 20% of cases. A diffuse infiltrative form may occur with intraconal, muscular, or perineural involvement. The involvement is rarely multifocal within the orbit (<5%). Bilateral involvement is seen in 25%, usually with higher grade lesions. The best diagnostic clue on imaging is a solid, homogeneously enhancing orbital tumor that molds to and encases orbital structures. Restricted diffusion within the lesion can be another useful imaging feature to lymphoma as a likely diagnosis. Lymphoma can be primary to the orbit or secondary to systemic disease. Secondary ocular involvement occurs in 2–5% of patients with advanced systemic non-Hodgkin’s lymphoma (NHL). However, lymphomas are the most common primary orbital tumors accounting for 55% of malignant orbital tumors in adults 60 years and older. The majority of NHL of the orbit and orbital adnexa are extra nodal marginal-zone B-cell lymphomas of mucosa-associated lymphoid tissue (23). Clinical manifestations of orbital lymphoma include painless proptosis and motility disturbances. Whole body staging is necessary after diagnosis and is most often done with FDG PET CT. Low-grade tumors respond well to radiation therapy while chemotherapy is necessary for high-grade or systemic disease (24).

Other primary orbital tumors involving EOMs simulating TAO are rare. Benign tumors like hamartoma, primary granular cell orbital tumors, rhabdomyomas, and liposarcoma have been described (12, 25, 26). Metastases from distant sites may occur with melanoma, breast cancer, gastrointestinal, and lung carcinoma. They can present as isolated or multiple masses within the EOMs, orbital fat, globe, or bony orbit. Majority of patients have a diagnosis of primary malignancy at presentation (27). Patients may present with diplopia, visual disturbance, proptosis, and movement restriction. All muscles could be involved (28).

### Orbital Pseudotumor or Non-Specific Orbital Inflammatory Syndrome

This has been described as the most common differential of TAO occurring in about 40% of cases (12). Our third patient presented with uniocular proptosis and diplopia, hypothyroidism, and right orbital edema with enlargement of inferior rectus muscle on MRI. His orbital biopsy showed mild inflammation only. He had good response to steroids but the swelling did not subside completely. One possibility is orbitopathy related to Hashimoto’s thyroiditis. Most euthyroid and hypothyroid patients with orbitopathy are TRAb-positive and they are diagnosed as having euthyroid or hypothyroid Graves’ disease. In our patient, TRAb was negative. Cases of orbitopathy have been reported in patients with Hashimoto’s thyroiditis with TRAb negativity (6, 29). In these patients, orbitopathy is likely related to the specific production of antibodies to orbital tissue other than TRAb. Alternatively, this patient could also have idiopathic orbital inflammatory syndrome if other secondary systemic disease or metastatic lesion is ruled out.

Idiopathic orbital inflammatory syndrome is an orbital inflammatory process of unknown etiology and is diagnosed by excluding other possible causes of exophthalmos. It was first described by Birch-Hirshfeld to describe an orbital syndrome clinically resembling a neoplasm but surgical pathology showing only inflammation. Over a period of time, many difficult to diagnose systemic inflammatory diseases and lymphomas were falsely classified under this category. With better imaging and diagnostic modalities, this disorder now denotes non-specific benign orbital inflammation without evidence of specific local or systemic cause (29). Good corticosteroid response is seen. It has varied clinical and radiologic presentation. It may present with inflammation and mass effect with symptoms and signs of pain, chemosis, proptosis, and motility restriction. Imaging usually shows a defined or poorly demarcated mass. It occurs in any age or sex group. Single muscle involvement is more common but involvement of bilateral muscles sparing the tendons very similar to Graves’ has been noted too (30). Biopsy is required to exclude other diseases. FNA is not helpful as inflammatory cells may be seen even with malignancy and not conclusive for this etiology. Histopathologic material is more informative. It is a self-limiting disorder most of the times. Based on the cellular, stromal, or vascular component, orbital pseudotumor is divided into histopathologic subclasses. These are sclerosing, granulomatous, vasculitic, and eosinophilic varieties. Therapy with steroids is advised during active phase as it rarely has a malignant course with extraocular muscle involvement and optic nerve damage. Steroid therapy classically results in rapid improvement, but cases unresponsive to steroids have been reported. In such cases, low-dose irradiation has been administered with varying success.

## Other Less Common Differentials

Other conditions, which may resemble TAO are vascular malformations, infections and neuromuscular dysfunction. Some vascular disorders causing myopathy are carotid cavernous fistulas and congenital orbital vascular malformations. In these disorders, arteriovenous shunting into cavernous sinus causes retrograde flow along superior ophthalmic vein causing venous distension and tissue edema with enlargement of muscles. Also, decreased pressure within the microcirculation leads to low tissue perfusion, hypoxia, and ophthalmoplegia (31). Orbital cellulitis from bacterial infections is more common, but uncommon infections like trichinosis, cysticercosis, and Lyme’s disease have been described. Neuromuscular disorders like myasthenia gravis cause extraocular muscle weakness, but not enlargement. Chronic progressive external ophthalmoplegia is an extremely rare condition where extraocular muscle size is reduced on imaging (32).

## Imaging

Imaging is very important and may give clues to diagnosis in atypical cases. Observation of muscle shape, size, borders, pattern of involvement, surrounding soft tissues, and bone involvement can help in the differential diagnosis (33, 34). In TAO, enlargement of EOMs and increase in retrobulbar fat volume is noted. The classical criteria is spindle-shaped enlargement of EOMs >4 mm without involvement of the tendon on both MRI and CT. Preferential muscle involvement, starting with rectus inferior followed by the medial rectus, the superior rectus, and finally the lateral is a specific finding, seen best on coronal views (33).

MRI gives better soft-tissue differentiation. T2-weighted images detect edema, inflammation in EOMs, and also help rule out other orbital pathology like infiltrative process. Orbital myositis is the most common etiology mimicking graves. It may involve any extraocular muscle including the tendons, may show medial bowing of the muscle with infiltration of fat. Inflamed muscles show low intensity on T1-weighted images and increased intensity on T2-weighted images. Tumors also have low-signal intensity on T1 and moderate to marked increase on T2 (34).

CT provides good imaging of orbital apex and optic nerve. In Graves’ orbitopathy, CT shows fusiform enlargement of the extraocular muscle, smooth borders, and non-involvement of tendons. Orbital fat is normal. Myositis on CT is characterized by contrast enhancement and likely diffuse irregular enlargement of muscles involving tendons in most cases. Orbital fat may be involved suggestive of inflammation. Concomitant periorbital findings may point occasionally to systemic disease. Malignancy may show single nodular muscle enlargement in most cases with sharp borders and occasional bone change. AV malformations may show homogenous enlargement of multiple EOMs with sharply defined borders and distension of superior ophthalmic vein. Infection and orbital cellulitis may show blurred muscle margins, adjacent sinus infiltrate, and orbital fat infiltration (35).

## Conclusion

Graves’ disease is the most common cause of orbitopathy. Hashimotos thyroiditis results in orbitopathy in a small percentage of patients. Alternate etiologies should be considered in the absence of hyperthyroidism and negative thyroid antibodies. Orbital imaging should always be performed. Other rare diagnoses like orbital pseudotumor, orbital lymphoma, orbital sarcoidosis, and orbital neoplasms should be evaluated for in the event of atypical clinical, biochemical, and imaging findings with an orbital biopsy. As vision loss can occur in some of these cases, accurate diagnosis, and treatment are imperative.

## Ethics Statement

Consent: written, informed consent was obtained from all patients mentioned in our manuscript both for the purposes of participating in the research as well as for their personal information to be published.

## Author Contributions

NB is responsible for coordinating with other authors and writing up the manuscript. MJ was responsible for writing Case 2 and part of the discussion as well as editing of manuscript. VW contributed to editing imaging findings and references. GB provided images and description for the same. FF is the final author who reviewed the manuscript and provided advise.

## Conflict of Interest Statement

The authors declare that the research was conducted in the absence of any commercial or financial relationships that could be construed as a potential conflict of interest. The reviewer, SF, and handling editor declared their shared affiliation, and the handling editor states that the process nevertheless met the standards of a fair and objective review.

## References

[1] Bahn RS Heufelder AE Pathogenesis of Graves’ ophthalmopathy. N Engl J Med (1993) 329(20):1468–75. 10.1056/NEJM199311113292007 8413459

[2] Wyse EP McConahey WM Woolner LB Scholz DA Kearns TP Ophthalmopathy without hyperthyroidism in patients with histologic Hashimoto’s thyroiditis. J Clin Endocrinol Metab (1968) 28(11):1623–9. 10.1210/jcem-28-11-1623 5754841

[3] Bahn RS Graves’ ophthalmopathy. N Engl J Med (2010) 362(8):726–38. 10.1056/NEJMra0905750 20181974 PMC3902010

[4] Dolman PJ. Evaluating Graves’ orbitopathy. Best Pract Res Clin Endocrinol Metab (2012) 26(3):229–48. 10.1016/j.beem.2011.11.007 22632361

[5] Daumerie C Duprez T Boschi A. Long-term multidisciplinary follow-up of unilateral thyroid-associated orbitopathy. Eur J Intern Med (2008) 19(7):531–6. 10.1016/j.ejim.2008.01.013 19013383

[6] Yoshihara A Yoshimura Noh J Nakachi A Ohye H Sato S Sekiya K Severe thyroid-associated orbitopathy in Hashimoto’s thyroiditis. Report of 2 cases. Endocr J (2011) 58(5):343–8. 10.1507/endocrj.K11E-019 21427503

[7] Bartley GB. The epidemiologic characteristics and clinical course of ophthalmopathy associated with autoimmune thyroid disease in Olmsted County, Minnesota. Trans Am Ophthalmol Soc (1994) 92:477–588. 7886878 PMC1298522

[8] Verma R Gupta M Mehta VK. Thyroid associated orbitopathy. BMJ Case Rep (2013) 2013:bcr2013009920. 10.1136/bcr-2013-009920 23737589 PMC3703025

[9] Khoo DH Eng PH Ho SC Tai ES Morgenthaler NG Seah LL Graves’ ophthalmopathy in the absence of elevated free thyroxine and triiodothyronine levels: prevalence, natural history, and thyrotropin receptor antibody levels. Thyroid (2000) 10(12):1093–100. 10.1089/thy.2000.10.1093 11201855

[10] Forbes G Gorman CA Brennan MD Gehring DG Ilstrup DM Earnest F IV. Ophthalmopathy of Graves’ disease: computerized volume measurements of the orbital fat and muscle. AJNR Am J Neuroradiol (1986) 7(4):651–6. 3088943 PMC8334661

[11] Nugent RA Belkin RI Neigel JM Rootman J Robertson WD Spinelli J Graves orbitopathy: correlation of CT and clinical findings. Radiology (1990) 177(3):675–82. 10.1148/radiology.177.3.2243967 2243967

[12] Lacey B Chang W Rootman J. Nonthyroid causes of extraocular muscle disease. Surv Ophthalmol (1999) 44(3):187–213. 10.1016/S0039-6257(99)00101-0 10588439

[13] Fallahi P Ferrari SM Ruffilli I Elia G Biricotti M Veta R The association of other autoimmune diseases in patients with autoimmune thyroiditis: Review of the literature and report of a large series of patients. Autoimmun Rev (2016) 15(12):1125–8. 10.1016/j.autrev.2016.09.009 27639841

[14] Baughman RP Lower EE Kaufman AH. Ocular sarcoidosis. Semin Respir Crit Care Med (2010) 31(4):452–62. 10.1055/s-0030-1262213 20665395

[15] So WL Hardy TG McKelvie P. Atypical clinical presentation of isolated extraocular muscle sarcoid. Case Rep Ophthalmol Med (2012) 2012:370258. 10.1155/2012/370258 23320221 PMC3540650

[16] Heiligenhaus A Michel D Koch JM Nodular scleritis in a patient with sarcoidosis. Br J Ophthalmol (2003) 87(4):507–8. 10.1136/bjo.87.4.507 12642326 PMC1771607

[17] Imes RK Reifschneider JS O’Connor LE. Systemic sarcoidosis presenting initially with bilateral orbital and upper lid masses. Ann Ophthalmol (1988) 20(12):466–7. 3218815

[18] Hoang TD Mai VQ Clyde PW Glister BC Shakir MK. Multinodular goiter as the initial presentation of systemic sarcoidosis: limitation of fine-needle biopsy. Respir Care (2011) 56(7):1029–32. 10.4187/respcare.01000 21352663

[19] Antonelli A Fazzi P Fallahi P Ferrari SM Ferrannini E. Prevalence of hypothyroidism and Graves disease in sarcoidosis. Chest (2006) 130(2):526–32. 10.1378/chest.130.2.526 16899854

[20] Grimson BS Simons KB. Orbital inflammation, myositis, and systemic lupus erythematosus. Arch Ophthalmol (1983) 101(5):736–8. 10.1001/archopht.1983.01040010736006 6847461

[21] Weinstein JM Koch K Lane S. Orbital pseudotumor in Crohn’s colitis. Ann Ophthalmol (1984) 16(3):275–8. 6712071

[22] Patrinely JR Osborn AG Anderson RL Whiting AS. Computed tomographic features of nonthyroid extraocular muscle enlargement. Ophthalmology (1989) 96(7):1038–47. 10.1016/S0161-6420(89)32785-0 2771351

[23] Payne JF Shields CL Eagle RC Jr Shields JA. Orbital lymphoma simulating thyroid orbitopathy. Ophthal Plast Reconstr Surg (2006) 22(4):302–4. 10.1097/01.iop.0000225422.69538.17 16855508

[24] Eckardt AM Lemound J Rana M Gellrich NC. Orbital lymphoma: diagnostic approach and treatment outcome. World J Surg Oncol (2013) 11:73. 10.1186/1477-7819-11-73 23506357 PMC3616859

[25] Purohit BS Vargas MI Ailianou A Merlini L Poletti PA Platon A Orbital tumours and tumour-like lesions: exploring the armamentarium of multiparametric imaging. Insights Imaging (2016) 7(1):43–68. 10.1007/s13244-015-0443-8 26518678 PMC4729705

[26] Cunniffe G Fernandez J Alonso T Balaguer O Dinares C Huguet P Thyroid orbitopathy obscuring the diagnosis of a rare neuromuscular hamartoma of the superior rectus muscle in an adult. Orbit (2010) 29(3):168–70. 10.3109/01676830903537146 20497087

[27] Spraul CW Lang GE Lang GK. [Orbital myopathy in metastatic malignant paraganglioma: a paraneoplastic syndrome?]. K lin Monbl Augenheilkd (1996) 209(2–3):153–7. 10.1055/s-2008-1035296 8992077

[28] Jaeger MJ Green WR Miller NR Harris GJ. Granular cell tumor of the orbit and ocular adnexae. Surv Ophthalmol (1987) 31(6):417–23. 10.1016/0039-6257(87)90033-6 3039674

[29] Grzesiuk W Szydlarska D Pragacz A Bar-Andziak E. Thyroid-associated orbitopathy in patients with Hashimoto’s thyroiditis: a case report. Pol Arch Med Wewn (2008) 118(5):318–21. 18619184

[30] Dresner SC Rothfus WE Slamovits TL Kennerdell JS Curtin HD. Computed tomography of orbital myositis. AJR Am J Roentgenol (1984) 143(3):671–4. 10.2214/ajr.143.3.671 6331757

[31] Kirsch E Hammer B von Arx G. Graves’ orbitopathy: current imaging procedures. Swiss Med Wkly (2009) 139(43–44):618–23. 19950023 10.4414/smw.2009.12741

[32] Muller-Forell W Kahaly GJ. Neuroimaging of Graves’ orbitopathy. Best Pract Res Clin Endocrinol Metab (2012) 26(3):259–71. 10.1016/j.beem.2011.11.009 22632363

[33] Mombaerts I Goldschmeding R Schlingemann RO Koornneef L. What is orbital pseudotumor? Surv Ophthalmol (1996) 41(1):66–78. 10.1016/S0039-6257(97)81996-0 8827931

[34] Sanders MD Hoyt WF Hypoxic ocular sequelae of carotid-cavernous fistulae. Study of the caues of visual failure before and after neurosurgical treatment in a series of 25 cases. Br J Ophthalmol (1969) 53(2):82–97. 10.1136/bjo.53.2.82 5773469 PMC1207139

[35] Carlow TJ Depper MH Orrison WW Jr. MR of extraocular muscles in chronic progressive external ophthalmoplegia. AJNR Am J Neuroradiol (1998) 19(1):95–9. 9432164 PMC8337335

